# Living with a spouse with chronic illness – the challenge of balancing demands and resources

**DOI:** 10.1186/s12889-019-6800-7

**Published:** 2019-04-23

**Authors:** Elisabet Eriksson, Maria Wejåker, Anna Danhard, Annika Nilsson, Marja-Leena Kristofferzon

**Affiliations:** 1 Department of Health and Caring Sciences, University of Gävle, Kungsbäcksvägen 47, 801 76 Gävle, Sweden; 2 Department of Public Health and Caring Sciences, Section of Caring Sciences, Uppsala University, Uppsala, Sweden

**Keywords:** Chronic illness, Partner, Resources, Formal care, Daily life

## Abstract

**Background:**

The number of partners providing informal care for their chronically ill spouse is rising, and they describe their daily life as demanding. The aim of this paper was to describe the partners’ experiences of living with a person with chronic illness and how they manage everyday life.

**Methods:**

A descriptive design with a qualitative approach was used. A purposive sample of 16 Swedish partners with a chronically ill spouse were interviewed. The interviews were recorded, transcribed, and analyzed using qualitative content analysis.

**Results:**

Four main themes were identified: ‘Managing challenges in daily life,’ ‘Seeking support and use own capabilities to manage life,’ ‘Appreciating the good parts of life’ and ‘Adapting to constant changes and an uncertain future’. Their experiences of support from formal care providers varied; they expressed the need for more assistance from the health care sector.

**Conclusions:**

The partners experienced many challenges in everyday life when providing informal care for their chronically ill spouse. This affected both their physical and psychological health, as they had limited time for themselves. The partners seemed to receive more support from their informal network than from formal care providers. In handling daily life, the partners balanced demands and resources to identify possibilities to move forward and find meaning in life. This is congruent with theories by Antonovsky, and Folkman and Lazarus that describes meaningfulness and how to handle challenges in everyday life.

## Background

Chronic illness or noncommunicable diseases, including cardiovascular diseases, cancers, respiratory diseases and diabetes, are the leading cause of death worldwide [1]. Chronic illness is a long-term condition that often progresses slowly and can seldom be completely cured. Characters of most chronic illnesses include complex causality, a long development period, sometimes without any symptoms, a time with increasing symptoms of ill-health and perhaps functional impairment [2]. Patients with chronic illness such as heart failure, multiple sclerosis and Parkinson’s disease need to manage symptoms, treatment, functional impairments [3–5] and unpredictable situations [6, 7]. Patients with chronic illness with high symptom burden affects not only the patient, but also the entire family. To maintain a normal life, partners with a chronically ill spouse are often involved in the spouse’s daily activities [8], and the partner’s ability to cope with the situation is stressed. Also, partners with a chronically ill spouse may themselves be older [9] and may have their own health issues and diseases, as older people are more likely to have chronic illnesses and multimorbidity [10]. In caregiving research, both positive and negative aspects of informal care is reported**.** Ray [11] found that partners in a long-term marriage, find it natural to provide informal care for their ill spouse. Partners in a happy marriage emphasized love and affection as motivators for providing care, whereas those in an unhappy relationship emphasized duty and obligation. Although it may be considered natural to assist one’s ailing spouse, providing care affects the caregiving partner in different ways. Limited time for own interests [12], negative effects on social life [13, 14], reduced quality of life [15] and experience of psychological distress [16, 17], are some consequences described in the literature.

Although, several studies focus on the burden for partners providing informal care, literature of positive aspects of caregiving is increasing. A literature review found how partners providing care to a spouse experienced positive emotions and personal growth [18]. Golics et al. [19] found that family members, including partners, experienced positive effects of the patient’s illness on their own life; such effects were described as improvement in family relations and appreciating one’s own life.

Regarding support for partners, informal caregivers’ situation has been recognized in recent years. Providing informal care to a spouse or family member should in a Swedish context be a voluntary commitment. Therefore, in 2009, there was a change in the Swedish Social Services Act, 5 Chap., § 10, which obliged municipalities to provide support for those who care for or support relatives who are chronically ill, elderly or who have a disability [20]. The aim is to reduce the family’s mental and physical stress. Support may take different forms depending on the needs of the relative, but includes information, individual and group discussions, education, help in the home or accommodations. However, the support caregiving partners receive from formal care providers differs. A report from the National Board of Health and Welfare [21] found that it is difficult to monitor and describe the impact of the new provision on individual relatives.

In light of the limited knowledge about the caregivers’ situation, in the current study, we seek to describe how partners to spouses with chronic illness manage everyday life, and how they find and use their personal resources to stay healthy. A theory that looks at human resources and not the focuses on barriers is therefore appropriate in this context. In Antonovsky’s theory of salutogenesis [22], health is described as a process in which the individual can move toward two poles, health and unhealthy. As the core concept, sense of coherence (SOC), consist of three dimensions: comprehensibility, manageability and meaningfulness. Comprehensibility refers to events that are understandable and predictable. Manageability refers to the belief that one has the ability to handle stressful situations and meaningfulness refers to the capacity to create and recreate meaning while managing life demands. To develop a strong SOC, the individual has to use general resistance resources. Thus, individuals with well-established SOC are more likely to handle stressful situations [22]. Lazarus and Folkman [23] further describe coping as a personal ability to manage stressful situations and can be divided into two groups. Problem-focused coping involves addressing the problem, whereas emotion-focused coping involves trying to reduce the negative emotions associated with the problem.

In an aging population [24], it is reasonable to suppose that more relatives will become informal caregivers. Further, because comorbidity exists and few patients have only one disease, it is an advantage to include a group with multiple ill health conditions. People find ways to live positively although chronic illness is characterized by a complex and unpredictable life situation. Thus, we need to know more about what is the common resources that partners to spouses with chronic illness have and which coping strategies they use. The aim of the study was to describe partners’ experiences of living with a spouse with chronic illness and how they manage everyday life.

## Methods

### Design

This study is part of a larger project, where one part was longitudinal with a quantitative approach and the other part cross-sectional with a qualitative approach [25, 26]. Here we present one study from the qualitative part [27, 28].

### Sample and setting

The sample was selected between June 2015 and January 2016 from the larger study [25], conducted in central Sweden. Purposive sampling was used to obtain variation in patients’ age, gender and chronic illness [27, 28]. The inclusion criteria for participating were that participants should understand the Swedish language, be living in a relationship with a person who has had a chronic illness > 3 months and that both patient and partner provide their consent to participate. The partners’ experiences are presented in the present paper.

Twenty-six couples were contacted by telephone and asked to discuss possible participation and 16 participated. Ten couples declined participation: two patients did not have any symptoms, one patient was too unhealthy and seven patients were not interested in participating. The medical diagnoses of the patients were: heart failure (*n* = 5), multiple sclerosis (*n* = 5), Parkinson’s disease (*n* = 3) and stroke (*n* = 3). The group of partners included seven men and nine women; their mean age was 70.5 years (53–88).

### Data collection

A semi-structured interview guide was developed. The authors formulated the main open questions based on the concept of SOC by Antonovsky [22]. Background variables such as family, work, and social network, how long the couple had lived together and the participants’ own health problems were also included. The participants were then asked to talk about how they feel, what a typical day looks like, what a working day might look like (if the person works), what their leisure time is like, how they handle stress in everyday life, what resources they have to manage everyday life, who provides support for dealing with everyday life, what makes life meaningful and what connection they experience with others in everyday life.

The interviews were conducted by AD and MW over a period of 8 months. The participants were informed about the study. They were asked to choose the place and time for the interview. The interviews were conducted in the participants homes and varied between 31 and 80 min, were audio recorded and then transcribed verbatim. The interviews were conducted in conversational form so that each interview was unique. Supplementary questions were asked such as: Could you tell us more? Two test interviews were conducted and feedback was provided by two members of the research team. One interview question was added to get a clearer perspective on the partners’ situation: “How has your spouse’s illness affected your daily life?” The test interviews were included in the data analysis.

### Data analysis

The interviews were read several times by the first author to get a sense of the whole and further analyzed using an inductive qualitative content analysis, as described by Graneheim and Lundman [29]. Each interview was seen as a unit of analysis. From the text, meaning units were extracted that comprised sentences or whole paragraphs. The meaning units were then condensed and given codes. The codes were subsequently compared and grouped based on similarities and differences, and later abstracted to create temporary subthemes and themes. As described by Graneheim and Lundman [30], subthemes and themes are recurrent threads of meaning in the text. The authors discussed and finally agreed upon the subthemes and themes. Open Code [31] was used to assist with data organization.

### Ethical considerations

The study was approved by the regional ethical review board in Uppsala (reg. no. 2010/346). The participants received oral and written information about the study. Written informed consent was obtained from all participants prior to the interview. Since data collection of the spouse with chronic illness and the partner took place at the same time by two researchers, spouses knew that the partner would talk about them.

## Results

### Sample characteristics

The participants were all in a long-term relationship and lived together, with the exception of one couple that was married but lived in separate apartments. All participants had living children (1–3 children), with the exception of one whose son had died recently; most of them had grandchildren. Thirteen participants were retired, two were still working and one was on sick leave and did not think he would return to work. Most of the participants had some physical limitations (Table 1). Because most of the informants were retired, they spent their time doing ordinary activities such as preparing meals, shopping, tidying up, taking walks and engaging in hobbies. Leisure activities consisted of visiting relatives and friends, going to the cinema or concerts, singing in a choir, doing photography, watching hockey and taking shorter trips.

**Table 1:** Characteristics of the participants (*N* = 16)

Variables	Participants (*N* = 16)
Gender	7 Male
	9 Female
Age (mean, SD, min-max)	70.5 (9.3, 53–88)
Marital status	11married
	5 lived together (38–60 yrs)
Living conditions	11 lived in a house
	5 lived in a flat
Health situation	2 described themselves as healthy
	7 described 1–3 diseases^a^
	9 described 1–3 symptoms^b^
	1unknown
Time of diseases/ symptoms	4–40 yrs
Important social networks	15 had children
	14 had grand children
	7 had contact with other relatives
	8 had contact with friends
	2 had contact with neighbors
	1 had contact with colleagues

^a^Diseases: heart disease, hypertension, diabetes, fibromyalgia, multiple sclerosis, anemia, herniated disc, cerebral hemorrhage, fibromyalgia, hydrocephalus, musculoskeletal pain

^b^Symptoms: back pain, knee pain, anxiety, tiredness, weakness, depressed mood, weight loss, reduced mobility, problem with balance

### Themes and sub-themes

The results are presented using nine sub-themes and the following four themes ‘Managing challenges in daily life,’ ‘Seeking support and use own capabilities to manage life,’ ‘Appreciating the good parts of life’ and ‘Adapting to constant changes and an uncertain future’ (Table 2). In the following section, each theme is described by its sub-themes with quotes from the interviews. Each interview was given an identification number.

**Table 2:** Themes and subthemes regarding partner’s experiences of living with a person with long-term illness

Themes	Subthemes
Managing challenges in daily life	Balancing leisure activities to suit the spouse’s health
	Adapting social life to the spouse’s health
	Taking over many practical things is a balancing act
Seeking support and use own capabilities to manage life	Trying to find support
	Taking advantage of resources and earlier experiences
Appreciating the good parts of life	Having relationships with family members and friends
	Being able to enjoy life values gives satisfaction
Adapting to constant changes and an uncertain future	Finding ways to accept the spouse’s illness Worrying about the future

### Managing challenges in daily life

This theme describes how the participants adjusted their own life in relation to the health of their spouse.

#### Balancing leisure activities to suit the spouse’s health

Experiences of limitations involved balancing daily activates in relation to their ailing spouse’s health. The participants described how they were limited in various ways because they did not want to leave their spouse alone for longer periods of time. One man said, *then it became very difficult…for me, 4 months, it was essentially 24-h attendance. And it was a difficult* (H101). Just as this man reported, some other participants as well experienced having very little time for themselves. It affected their own health when they could not exercise as much as they wanted to; they took fewer walks, played golf less often, did not go swimming or to the gym. There was also an expressed wish to travel more, but this had become difficult because they could not take their wheelchair-bound spouse everywhere or because they needed to plan trips that would not be too tiring for their spouse.

#### Adapting social life to the spouse’s health

According to the participants, the health of their spouse influenced their social life and determined whether they visited friends or invited someone over. Some felt they had isolated themselves because they were too tired to socialize with others, even though they wanted to. This situation created feelings of loneliness and isolation. One woman said, *I don’t need to isolate myself, but it’s after all, because sometimes he requires so much* (H308). The participants mentioned that, given their age, it was natural to have fewer friends, but at the same time, some felt they were not being invited to social gatherings like they had before. They thought their friends had tired of inviting them, knowing they could not come because of their spouse’s condition.

#### Taking over many practical things is a balancing act

As the spouse’s functional abilities had declined over time, the participants had taken over more everyday activities, such as driving, shopping, cleaning and cooking. They found it natural to carry out more activities, but also challenging, as they had to learn new things. For example, some male participants had learned more about cooking, and some female participants took over gardening and paying bills. However, more practical work was also described as demanding when they had to assume more responsibility for their spouse’s care and treatment. One woman said, *one is very much involved in everything… because he has a little dementia too, I’m the one who handles all the hospital appointments and everything, all the doctors and all the contacts like that and the medications (P169).* The participants described how they tried to balance between helping their spouse and letting their spouse do the things he/she could still do. This balancing act was complicated, because it could change from day to day depending on their spouse’s condition.

### Seeking support and use own capabilities to manage life

Because the participants experienced both physical and psychological challenges due to their spouse’s illness, they looked for support and encouragement within themselves and from others.

#### Trying to find support

Experiences of living with the spouse’s illness involved finding formal and informal support for the present situation and using one’s own resources to manage daily life. Formal support came from health care providers such as physiotherapists, counselors, nurses and doctors. They received various kinds of support, such as information about their spouse’s disease. One spouse was able to stay at a daycare center when the participant needed some time on her own. Another participant described the freedom she and her spouse experienced once he was given an electric wheelchair. However, experiences of support from the health care system differed. While some were satisfied with the support, others were disappointed, for example with hospital staff. One woman said, *But I thought when you come to a university hospital, you’d think it would be great, but it wasn’t (P169).*

The informal support came from their family, friends, neighbors, patient associations, church and present or former work colleagues. Family members provided both material and emotional support. The participants felt that they could call their children when they needed help or just wanted to talk. One man said when he was sick himself, *When I was hospitalized then she (daughter) was at home with (wife)...She took off from work and she was home with (wife). And then she visited me at the hospital* (H253). However, some also expressed wanting more support from their children, both with practical issues and emotional support, for example more frequent phone calls or visits. Participants also mentioned that their children did not fully understand their situation and that they felt alone. When the participants needed encouragement, most of them felt that they could talk to their spouse and that they encouraged and supported each other. Planning and making shorter or longer trips together was another way of finding encouragement together. Among the participants, there was one deviant case, in which the relationship was very strained. The participant, who was ill with the same chronic disease, felt it was only the patient, who received support from both health care providers and from their friends. This led to feelings of being neglected, and forgotten by the community.

#### Taking advantage of resources and earlier experiences

Earlier experiences of success in life or of being optimistic served as resources that the participants used to manage daily life. As one man said, *I’m a pretty positive person…I don’t let the situation get the better of me* (S19). Having previous experience of being able to handle difficult situations at work or in family life gave the participants hope that they would also manage now. Being in their relationship for many years was also described as a resource. Knowing what the spouse liked was said to reduce the need for talking. However, communication was also described as crucial to the relationship, *“We try to talk more, not less. For you need to talk about things to lighten them up, otherwise. Because there’s always something to take care of.”* (S15). The participants also mentioned how they wanted to get along with their spouse for their own sake. They had jointly decided to wait with receiving practical help from the primary care services until they really needed it.

When times were difficult, for example when their spouse had severe pain, was feeling down or was less mobile, the participants did not always ask for assistance from others, but handled the situation themselves. They described how they reasoned with and talked to themselves. In this connection, one man said, *then I try to discuss things with myself* (H101). Taking a break apart from the partner and going out for walks, engaging in sports or a hobby were other ways of managing difficult times. According to the participants, they and their spouse felt better when they went on shorter or longer outings together, and therefore they encouraged their spouse to go with them.

### Appreciating the good parts of life

Under this theme, the participants described the importance of being able to enjoy and find meaning in life.

#### Having relationships with family members and friends

Being able to appreciate close relationships was considered one of the good parts of life. The participants described how pleasing relationships with their spouse, children, siblings and grandchildren had helped them appreciate life. *Having loved ones, of course. It’s very important, you notice it when you’ve been affected in this way. How important it is* (P98). Apart from family members, warm relationships with friends, acquaintances and neighbors were experienced as important. Others had close relationships with other members of the church or the bridge club.

#### Being able to enjoy life values gives satisfaction

The participants felt that having good health was vital, *being healthy ... that’s what’s ... well maybe not the meaning of life, but life is much easier if you’re healthy* (MS62). Enjoying good health meant that the participants could continue with their hobbies, spend time in the garden, walk to the shop or have a nice time together with friends. Life values included being able to help their spouse with practical issues so that he/she could be self-reliant as long as possible. Moreover, the participants mentioned how they appreciated traveling together or just having a nice time together as a couple.

### Adapting to constant changes and an uncertain future

This theme represents how the participants had accepted their spouse’s illness, but also how they had distanced themselves from this situation. It was evident that although the participants received support, as shown above, they sometimes dealt with the situation themselves.

#### Finding ways to accept the spouse’s illness

The participants found different ways to accept their current situation. Some accepted the spouse’s illness, and it worked out well for them. It was common to relate their living situation to old age, as one husband said, *We’ve both gotten to the age, so we’re fully aware that everything might not be like before... it’s well ... almost a sort of acceptance* (S19).

Some participants had confidence in their ability to handle the situation, and others thought it would work out in the future. As new problems arose, the participants found it natural to identify practical solutions.

#### Worrying about the future

The spouse’s disease also generated worries about both the present and the future, and some participants described how they sometimes cried or felt angry and expressed mental pain. One woman described her frustration when her husband, who had breathing difficulties, waited to go to the hospital or call for an ambulance until his condition was critical, *I don’t know how many times he’s been on the verge of dying, it’s quite unbelievable. At the same time as you get scared, and very angry, you still have to wait* (H159). Thinking about the future, the participants were uncertain about how things would be. Knowing that their spouse’s health will get worse, but not knowing when, raised many questions. They thought about how long they would be able to stay in their house or how long they could manage on their own.

Although the participants accepted the spouse’s illness, they were unsure about how to handle their situation. Some distanced themselves from it all, mentioning that they did not think so much about the illness. They tried to live like normal, because this had a value in itself. However, it was not obvious to all participants how they practically handled their circumstances, w*hat do I do, I don’t know, I don’t do anything special* (H159).

## Discussion

The present findings illustrate how the participants, who were living with a chronically ill spouse, experienced both physical and psychological challenges in everyday life. The challenges concerned keeping up with increased household duties and staying healthy, because they had limited time for exercise and for themselves. Further, the participants experienced challenges when they wanted to maintain their social life. To manage everyday life, the participants appreciated their own health and looked for support for themselves and their spouse. Also, having close relationships with family members and friends brought meaning and satisfaction in life, although daily life was dependent on the health of their spouse. The participants both accepted and distanced themselves from the constant challenges of everyday life. In this way, they found themselves balancing available resources and demands in order to handle daily life which is also confirmed by studies from other contexts than Sweden [32, 33]. This is also congruent with Antonovsky’s [22] and Folkman’s [23] and Lazarus’ theories.

The present study shows that the participants had limited time on their own. This affected their own physical health because they did not exercise as they used to. It also meant they could not continue with hobbies or sports to the extent they wished. This is in line with previous research about partners limited private life when they care for an ill spouse [11, 12, 34]. In the present study, the participants’ social life was affected and their living space was shrinking as they adapted to their spouse’s health. They expressed feelings of loneliness and isolation, although they wanted to spend more time with others. Similar findings have been reported in other studies, Golics et al. [19] found that family members’ social life was affected by having an ailing relative, and Sanders and Power [8] found that it was good for the couple when the partner could plan social activities. Regarding support, some participants reported having received support and were quite satisfied with it, others were disappointed. One participant, also with a chronic illness, felt neglected and forgotten by both formal health care providers and friends. Thus, the partner’s situation and needs may be disregarded by both professional health care workers and the surrounding community, family, and friends. In the context of nursing care, Boland et al. [35] stressed the need for rehabilitation services staff to make plans together with both the patient and the partner. Further, Miller et al. [36] suggested that working methods are needed to assess the situation of partners of a patient living with chronic illness, the goal being to promote behavior change and achieve positive pain treatment outcomes.

Informal support from family, friends, neighbors, a church and associations was important to the participants in the present study. They received both emotional and practical support from their informal network and they knew from where or from which organization they could receive the support they needed. For example, participants turned to friends for emotional support and assistance with practical tasks from neighbors and family. Some, however, mentioned wishing they received more support from their children. In the present study, being in the relationship for several years motivated the participants to continue supporting their spouse in spite of the illness. They reported that being able to help their spouse in the home and managing as a couple were meaningful to them. Finding meaning while providing informal care is also reported by others [14, 37]. The long relationship itself was described as resource. The present findings relate to Antonovsky’s [22] perspective on health in several ways. First, the participants in the present study run the risk of moving toward the unhealthy pole if they cannot exercise, which is essential to maintaining one’s health. Secondly, how the component of comprehensibility can be used to find ways to manage new situations. This also confirm how the components of comprehensibility and manageability of the daily life may help partners find meaning in life. In the present study, the participants reported receiving more support from their relatives and friends than from the health care sector. However, they did not receive sufficient attention from health care professionals, as pointed out in a Norwegian study where family carers lacked acknowledgement for their caring role by professional caregivers [37]. A Swedish study among informal care givers suggested that support need to be individualized and flexible to promote caregivers own health [14]. Formal caregivers need to ask about and follow up on the health of not only the chronically ill patient seeking care, but also his/her partner. Furthermore, health care personnel need to provide partners with information about available support from formal care providers such as formal centers for informal caregivers. Example of support could be time off from the caregiving tasks and encouragement from peer support groups.

To uphold normality and manage everyday life, the participants took over many household tasks, which has been reported by others [8, 11, 35] . The participants described it as natural and positive, in that they had learned many new things. However, the trick was to not take over too many household tasks – to know when to step in and help their spouse and when to let the spouse do what he/she could do. In these new situations, coping strategies were required to balance demands and resources in daily life. In relation to Folkman’s and Lazarus’ [23] theory, the partners applied both problem- and emotion-focused coping as well as meaning-based coping [38], when they adapted to the new demands. The participants used the strategy *positive reappraisal* when trying to find meaning in their new situation, and they revised their goals and planned activities based on the health of their spouse. Thus, they attempted to maintain a sense of purpose in daily activities and to maintain control, even though the health of their spouse was constantly changing. In this way, the participants’ ability to cope also nourished their hope, the hope to continue managing daily life as long as possible [39]. Boland et al. [35] reported that a complex interplay of emotions and coping strategies arises between a person with multiple sclerosis (MS) and those close to him/her. This relates to the present study, where some of the participants could relate to the influences of MS in their daily lives. A study by Lubre-Puerto et al. [34] suggested that informal caregivers from countries with a better economic situation may have more access to formal care and support and rate their ability to cope higher than do informal caregivers living in poorer countries. In the present study, participants’ experiences of everyday life relate to both Antonovsky’s [22] concept of sense of coherence and Lazarus’ and Folkman’s [23] theory of coping. How partners manage everyday life may depend on their own personal resources such as earlier experiences, health, how the relationship to the spouse functions, what support they are offered and the support partners and their spouse choose to receive.

### Methodological considerations

To strengthen credibility, participants were selected who varied in age, gender, former occupation and whose spouses had different chronic illnesses. Two pilot interviews were performed to test the feasibility of the interview guide. Further, excerpts from the transcribed text have been presented to strengthen credibility. To ensure dependability, only two persons conducted all the interviews. By using the interview guide, the same content areas were discussed with all participants. Two participants were very ill themselves, which could have influenced the interview situation in that they talked more about themselves than about their experiences as a partner. However, the other interviews were very extensive and the authors decided not to compensate for the two thinner interviews. For unknown reasons, seven patients were not interested in participating in the study. This could have affected the results of the partners, especially if these patients were severely ill. To enable transferability, a thick description of the participants’ demographic characteristics has been provided, along with details about data collection and the analysis process. However, transferring the results to other contexts should be done with caution as the study was based on a small sample size from a Swedish context.

## Conclusion

The present study, from a Swedish context, confirm earlier findings with regard to how the partners cope with challenges and how their own physical and psychological health is affected. To manage everyday life, partners adjust to the constant challenges and seek support from their informal network as well as from formal care providers. Our findings confirm the work of Antonovsky’s perspective of health, and Folkman’s and Lazarus’ theory of coping.

Future research need to develop methods for preventing physical and physiological ill health among partners with a chronically ill spouse. Formal care providers must acknowledge partners’ needs, develop evidence-based assessment guidelines, and provide efficient support to partners with a chronically ill spouse.

## Abbreviation

SOC: Sense of Coherence
